# Lentivirus-mediated Inhibition of Tumour Necrosis Factor-α improves motor function associated with PRDX6 in spinal cord contusion rats

**DOI:** 10.1038/srep08486

**Published:** 2015-02-16

**Authors:** Xiao Zhang, Lan-lan Shi, Xia Gao, Di Jiang, Zhan-qiong Zhong, Xi Zeng, Ying Rao, Xi Hu, Tian-zhi Li, Xiu-juan Li, Lei Li, Jian-min Chen, Qingjie Xia, Ting-hua Wang

**Affiliations:** 1 Center for Experimental Technology of Preclinical Medicine, Chengdu Medical College, Chengdu 610083, Sichuan, China; 2 Institute of Neuronscience, Kunming Medical University, Kunming 650031, China; 3 Institute of Neurological Diseases, Translational Neuroscience Center, West China Hospital, Sichuan University, Chengdu 610041, China

## Abstract

The recovery of motor function in rats is inhibited following contusion spinal cord injury (cSCI). However, the mechanism of tumour necrosis factor α (TNF-α) in motor function after cSCI associated with peroxiredoxin 6 (PRDX6) remains unknown. We randomly divided rats into four groups: sham, cSCI, vector and lentivirus mediating TNF-α RNA interference (TNF-α-RNAi-LV) group. The Basso, Beattie, Bresnahan (BBB) scale was used to evaluate motor function. Real-time quantitative PCR (qRT-PCR) and western blotting were used to detect the expression of TNF-α and PRDX6, which were located in neurons using immunohistochemistry (IHC) and immunofluorescence. Subsequently, lentiviral-mediated TNF-α was used to determine the role of TNF-αand the relationship of PRDX6 and TNF-α in cSCI. After cSCI, the motor capability of hind limbs disappeared and was followed by recovery of function. IHC analysis indicated that TNF-α and PRDX6 were primarily located in spinal cord neurons. TNF-α interference significantly improved neural behaviour and increased expression of PRDX6. Our study suggests that inhibition of TNF-α can promote the recovery of motor function. The underlying mechanism of TNF-α-promoted motor function may be connected with the up-regulation of PRDX6. This provides a new strategy or target for the clinical treatment of SCI in future.

Spinal cord injury (SCI), a type of serious trauma of the central nervous system, can eventually lead to motor and sensory dysfunction below the level of injury^1^,^2^. It contains the primary injury and secondary injury, which causes a series of variation in pathophysiology significance, such as inflammation, haemorrhage, oedema, apoptosis and oxidative stress^3^,^4^,^5^. Among these, inflammation plays an important role in secondary injury. Proinflammatory factors and cytotoxic factors, such as IL-1β,IL-6 and TNF-α, are the main causes of aggravated damage in cSCI^6^,^7^,^8^. Interventions for SCI treatment via regulating cytokines or an active oxygen strategy, are also unknown.

TNF-α, an inflammatory factor produced by astrocytes and microglia after SCI^9^,^10^,^11^,^12^,^13^, together with IL-1 and IL-6β, are demonstrated to play crucial roles in inducing neuronal apoptosis that affect sensory and motor function after SCI^14^. However, the mechanism of TNF-α influence in SCI has not been clear. Antioxidant enzymes, known as peroxiredoxins (PRDXs), consist of six members, namely PRDX1-6^15^. They are extensively expressed in prokaryotes and eukaryotes, which have very similar oxidative stress response of systems^16^,^17^. PRDX6 is a type of bifunctional enzyme with both peroxidase activity and Ca^2+^-independent phospholipase A2 (iPLA2) activity to protect against oxidative stress and to prevent cells from peroxidation leading to membrane injury^18^,^19^,^20^. Nigar reported that PRDX6 can attenuate retinal ganglion cell death by limiting reactive oxygen species (ROS) levels and maintaining Ca^2+^ homeostasis^21^. In addition, PRDX6 expression is down regulated upon serum deprivation and subsequently induced in a time-dependent manner in response to KGF, dexamethasone and H_2_O_2_^22^. Nevertheless, there have been few studies concerning PRDX6 in SCI. Moreover, the relationship between TNF-α and PRDX6 in SCI has yet to be determined.

In this study, we used the lentivirus-mediated RNA interference to suppress the expression of TNF-α (TNF-α-RNAi-LV) and attempted to find the possible association of TNF-α and PRDX6 and explore its mechanism in SCI. We aimed at providing new targets for the clinical treatment of SCI.

## Methods

### Animal

The experiments were conducted with a total of 282 adult female Sprague-Dawley (SD) rats (Dashuo of Laboratory Animal Co., Ltd, Chengdu), aged 12 weeks, with a body weight of 210 ± 10 g. They were randomly divided into groups of varying number for the following procedures.

### Animal model

Contusion spinal cord injury (cSCI) models were produced using a modified weight-dropping device. Briefly, after the animals were deeply anaesthetised with sodium pentobarbital (45 mg/kg, i.p.), they were fixed in a supine position and underwent a partial laminectomy of T9. A contusion cSCI was produced using a patented effect cSCI device (Patent number: CN200820141443) with an impact force of 3 × 10^−3^ N (0.10 kg × 0.03 m × 9.8 N/kg). The sham group and vector group animals underwent identical surgical dissection and exposure, but did not undergo injury to the spinal cord.

After the surgery, the rats were maintained in an isothermic cage until their recovery. Then, they were transferred to a bacteria-free biologically clean room set on a 12-hour light/dark cycle and provided with food and water. The bladders of the injured animals were manually emptied twice daily until normal function returned.

The usage of animals and experimental protocol in the ethics has been confirmed by a named institutional and licensing committee of ethics in Sichuan University. All experimental procedures were conducted in accordance with the guidelines outlined in the National Institute of Health (NIH) Guide for the Care and Use of Laboratory Animals (1996) and were approved by Institute of Neurological Disease, Translational Neuroscience Center, West China Hospital, Sichuan University.

### Construction of TNF-α lentiviral expression vector

The shRNA fragment with the highest interference efficiency was provided by GeneCopoeia (Guang Zhou, China) and the TNF-α lentiviral expression vector used to inhibit TNF-α was constructed. After this, TNF-α expression vector (5 μg) and viral packaging vectors (1 μl, GeneCopoeia, GuangZhou, China) were co-transfected into 293 cells to produce lentiviral particles, TNF-α-RNAi-LV. The viral supernatant was harvested at 48 hours post-transfection and filtered through a 0.45 μm cellulose acetate filter. Then, the 5 ml cell supernatant containing lentivirus was centrifuged (3500 g, 25 min). The precipitate was re-dissolved in 500 μl PBS. Finally, the lentivirus was frozen at −80°C.

### Injection of TNF-α-RNAi-LV and vector in to spinal cord in vivo

Injection of TNF-α-RNAi-LV and vector into the spinal cord in vivo was performed within 48 hours before the cSCI. Briefly, the rats were deeply anesthetised with sodium pentobarbital (35 mg/kg), fixed in a supine position, and then underwent a partial laminectomy to expose T9 of the spinal cord. An equal volume of lentiviruses (5 μl per rat) was injected into two points on a coronal plane of the T9 segment of the spinal cord. The animals were maintained at the optimum temperature and housed in comfortable cages.

### Neurobehavioral assessment

Hind limbs motor function recovery was assessed using the Basso, Beattie, and Bresnahan (BBB) motor rating scale^23^ every day from Day 1 to Day 28 after cSCI.

The animals were placed in a 1 m × 1 m field and allowed to move freely. The ipsilateral hind limb motor functions were rated with a 21 point scale. The measures were performed prior to surgery and at daily intervals thereafter.

According to the BBB scoring system, neurobehavioral assessments were performed by five people mastering the experimental criteria but did not know the groupings. Four colleagues acquired points, one acted as a quality control (remove one score of four colleagues) and data recorder. Every rat was assessed over 4 minutes and scores were obtained from the three of four evaluators. The mean score was recorded as the final result.

### Preparation of paraffin sections and specimens

Once deep anaesthesia was achieved, transcardial perfusion for the models was performed at the corresponding survival time point. According to the requirements of the different experimental techniques, models were also divided into morphological staining groups, sham group (n = 6), cSCI group (n = 6), vector group (n = 6), and TNF-α-RNAi-LV group (n = 6).

The former was firstly rinsed quickly with 0.9% normal saline (4°C), then slowly pre-fixed with 4% paraformaldehyde (pH = 7.3, 4°C). After 48 h, post-fixed specimens (T9s were removed) were dehydrated, then embedded in paraffin and sectioned (5 μm); the latter was directly rinsed quickly with 0.9% saline (4°C) for samples (1 cm long injury segment) and then preserved at −80°C.

### Immunohistochemistry

Immunohistochemistry was performed using the SP Kit (Zhongshan Jinqiao Biotechnology Co. Ltd.) method. After sections were dried, dewaxed, hydrated and underwent high-pressure antigen repairing. Sections were incubated in 3% hydrogen peroxide (filtered 0.01 M PBS preparation) at 37°C for approximately 10 min and then incubated in 5% sheep serum (filter 0.01 M PBS preparation) at 37°C for 25 min. Subsequently, anti-TNF-α (mouse, 1:150, Abcam) and anti-PRDX6 (rabbit, 1:150, EPITMICS) antibodies were incubated overnight at 4°C. Sections were then washed with 0.01 M PBS according to SP kit instructions, then sequentially incubated with reagent B (37°C incubating for 15 min) and reagent C (37°C incubating for 15 min) at intervals with 0.01 M PBS. Then, diaminobenzidine (DAB) was used to develop the stain and 0.01 M PBS was used to terminate the reaction. The sections were then subjected to haematoxylin staining, separation, transblue, and dehydration. Sections were made transparent, and mounted. They were observed with a light microscope (Motic inverted microscope) with a computer-assisted video camera.

### Immunofluorescence

After tissue was dewaxed, hydrated, high pressure antigen repaired and 5% sheep serum (filter 0.01 M PBS preparation) blocked 37°C for 25 min, Anti-TNF-α (Mouse, 1:100, Abcam) and anti-PRDX6 (Rabbit, 1:150, EPITMICS) were added overnight at 4°C. After they were washed, sections were incubated with fluorescence secondary antibody IgG (anti-rabbit Cy3: green, 1:100, Jackson)/IgG (anti-mouse 488: red, 1:200, Invitrogen) for 2 hours at 37°C. After DAPI mounting, the slides were observed using fluorescence microscopy. For double labelling, anti-GTAP/anti-NEUN was chosen to adapt to species of anti-TNF-α/anti-PRDX6.

### Western blotting

The spinal cord segments at rostral thoracic vertebrae 9 were obtained from the model group, specifically including sham group, 12 hour post operation (h), 1 day post operation (dpo), 3 dpo and 7 dpo.

After carefully removing the spinal cord meninges, spinal cord segments were homogenised on ice in a lysis buffer containing 0.05 M Tris-HCL (pH 7.4, Amresco, Solon, OH, USA), 0.5 M EDTA (Amresco), 30% TritonX-100 (Amresco), NaCl (Amresco), 10% SDS (Sigma, St Louis, MO, USA), and 1 mM PMSF (Amresco) and then centrifuged at 12000 r/min for 30 min. The supernatant was obtained and stored at −80°C for later use. A 20 μl sample was loaded onto each lane and electrophoresed on 12% SDS-polyacrylamide gel (SDS-PAGE) for 2.5 h at a constant voltage of 120 V. The proteins were transferred from the gel to a nitrocellulose membrane blocked with PBS containing 0.05% Tween-20 (PBST) with 10% non-fat dry milk overnight at 4°C. The membrane was rinsed with PBST and incubated with the primary antibodies for anti-TNF-α (1:1000) and anti-PRDX6 at 4°C. The membrane was incubated with a HRP-conjugated goat anti-rabbit IgG (1:1000; Vector Laboratories, Burlingame, CA, USA) for 2 h at 37°C and was developed with an ECM kit and exposed against to X-ray film in a darkroom. Densitometry analysis for the levels of TNF-α protein was performed using the Bio-Gel Imaging system equipped with Genius synaptic gene tool software. β-actin (primary antibody, 1:200; secondary antibody, 1:5000; Santa Cruz Biotechnology, Inc., Santa Cruz, CA, USA) was used as an internal control.

### Real-time Quantitative PCR detecting System

The T9 segments of the spinal cords were obtained from the model group. Total RNA was extracted from spinal cord tissue of rats using Trizol reagent (Invitrogen) according to the manufacturer's protocol and reversed transcription to cDNA with the First Strand cDNA Synthesis kit (TaKaRa Biotechnology, Dalian, China). qRT-PCR was then performed to determine the expression of genes. For qRT-PCR, the primer sequences were as shown in table 1. qRT-PCR was performed in a DNA thermal cycler (ABI 7300) according to the following standard protocol: one cycle of 95°C for 2 min; 40 cycles of 95°C for 15 s, annealing for 20 s, and 60°C for 40 s. Relative expressions were calculated with normalisation to β-actin values by using the 2^−ΔΔCt^ method.

### Statistical analysis

All data are expressed as the means ± SEM. The numbers of positive cells were compared using one-way analysis of variance (ANOVA). The significance of the difference between the groups was calculated followed by Fisher's least significant difference post hoc test. Probability values (P) of less than 0.05 were considered to represent significant differences.

## Results

### Effect of motor function and changes in gene expression of TNF-α and PRDX6 after cSCI

**Figure 1:**
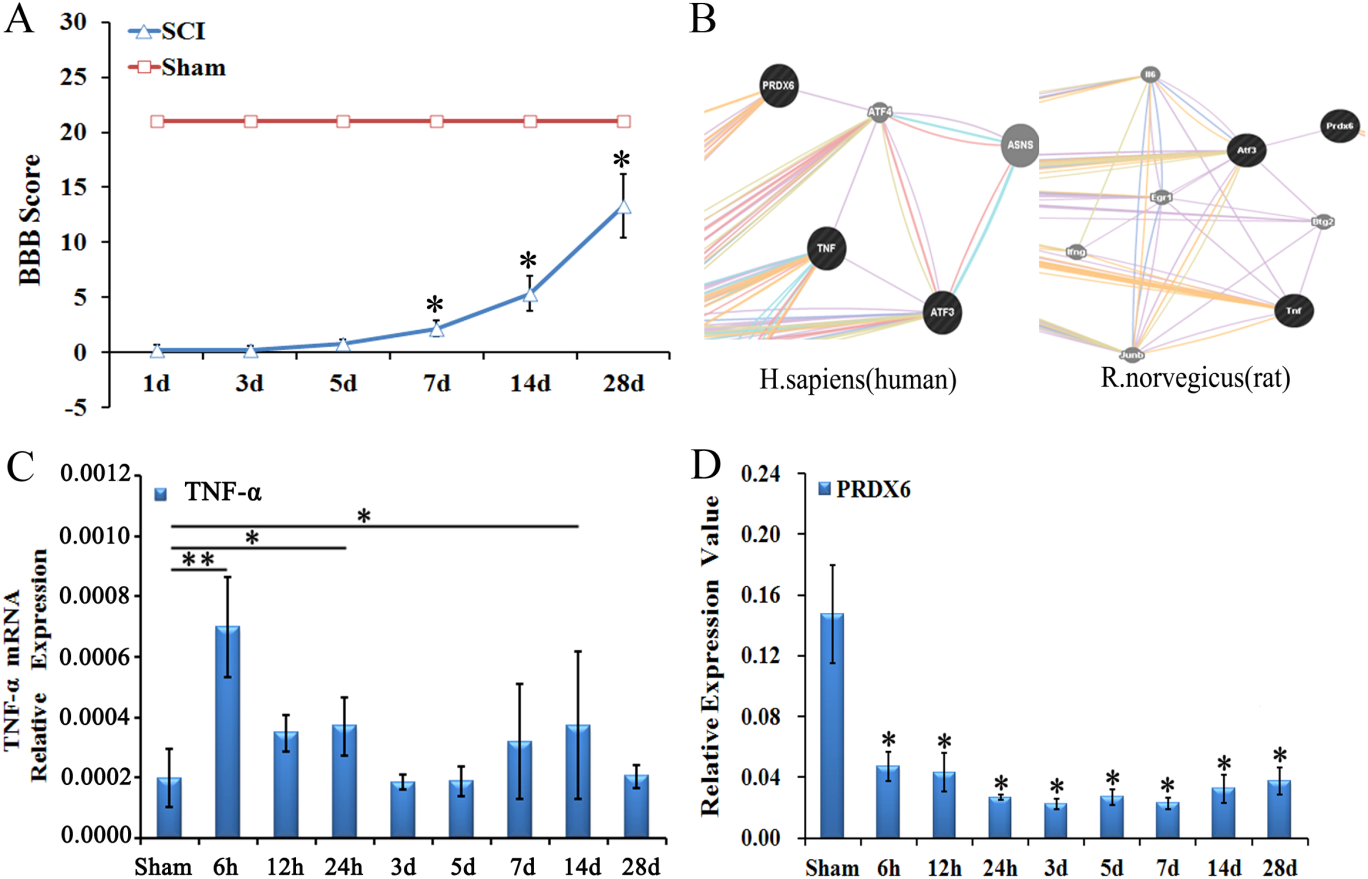
The change of BBB scores and the expression of TNF-α and PRDX6 after cSCI. (A) The BBB scores of rats with spinal cord contusion. Compared to the sham group, the BBB scores are significantly lower at each time point in the contusion group. The BBB scores increase at 7 dpo and remained so until at least 28d in the SCI group. (B) Bioinformatics show TNF-α and Atf3 are co-expressed, whereas PRDX6 co-expressed with Atf4. At the same time, Atf3 and Atf4 have the same structural domain, thus TNF-α might be associated with PRDX6. (C) The expression of TNF-α mRNA is significantly increased at 6 h, 24 h and 14 d after cSCI in the rats. (D) The level of PRDX6 mRNA is detected using qRT-PCR, which significantly decreased at 6 h, 12 h, 24 h, 3 d, 5 d, 7 d, 14 d and 28 d after cSCI. Values are expressed in mean ± SED * p < 0.05 *vs.* sham group.

The recovery of hind limb function following spinal cord contusion in rats was recorded using the BBB scale. Rats exhibited immediately paraplegia with no hind limb movement after surgery compared to the sham rats, obtaining a BBB score of 21. After spinal cord contusion, the rats exhibited significant motor dysfunction in hind limbs on 1 dpo and 3 dpo compared with the sham rats (P < 0.05). BBB score increased significantly on 7 dpo and 14 dpo compared to 3 dpo (P < 0.05). However, no further increase in recovery of motor function was observed 28 dpo. (Fig. 1A)

Molecular network prediction methods were used to detect whether there is a relationship between TNF-α and PRDX6. The results showed that TNF- α and ATF4 are co-expressed, PRDX6 and ATF4 are co-expressed, TNF- α and ATF3 are co-expressed, and ATF4 and ATF3 have physical interactions^29^. Another experiment showed that PRDX6 and ATF3 were co-expressed^30^ (Fig. 1B).

The expression of TNF-α and PRDX6 mRNA was detected using real time quantitative PCR, Compared to the sham group, the level of TNF-α mRNA was significantly increased 6 h, 24 h and 14d after cSCI in the rats (P < 0.05) (Fig. 1C). Comparatively, the level of PRDX6 mRNA was detected using qRT-PCR, which significantly decreased at 6 h, 12 h, 24 h, 3 d, 5 d, 7 d, 14 d and 28 d after cSCI when compared to those of the sham group (P < 0.05, Fig. 1D).

### The expression and localisation of TNF-α and PRDX6

**Figure 2:**
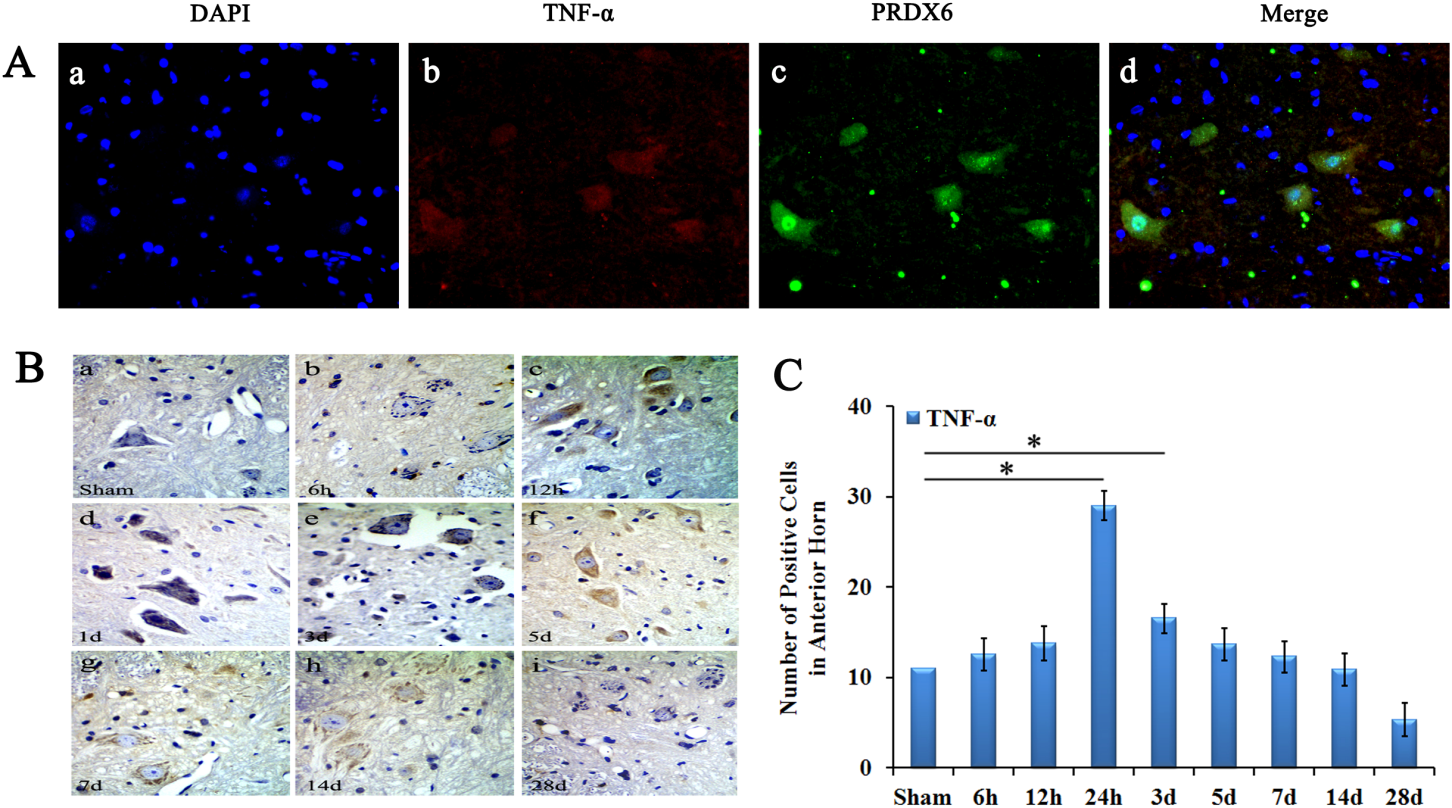
The location of TNF-α and PRDX6 following cSCI. (A) Localisation of TNF-α and PRDX6 in the spinal cord. DAPI (blue) stains the nucleus, TNF-α (red), PRDX6 (green) and merged images of spinal cord neurons and neuroglial cells after cSCI. TNF-α and PRDX6 are co-localised in neurons and neuroglial cells of the anterior horn of the spinal cord. Magnitude:40 × 10 μm (B) Immunohistochemistry shows that TNF-α was located in the cytoplasm of neurons of anterior horn in spinal cord. (C) The number of TNF-α-positive cells was significantly increased at 24 h and 3 dpo after spinal cord injury. Magnitude:40 × 10 μm. Values are expressed in mean ± SED * p < 0.05 *vs.* sham group.

Immunofluorescence was used to observe the location of TNF-α and PRDX6 in the rats with spinal cord injury. After cSCI, TNF-α was observed in spinal cord grey matter neurons and in neuroglial cells. TNF-α and PRDX6 was evidently co-localised in spinal cord neurons and neuroglial cells in the anterior horn of spinal cord (Fig. 2A).

The TNF-α-positive cells were located in the cytoplasm of neurons of the anterior horn in the spinal cord (Fig. 2B). The number of TNF-α-positive cells in grey matter were significantly increased at 24 hour and 3 dpo after spinal cord injury, then decreased and remained until 28 dpo (Fig. 2C).

### The protein expression of TNF-α and PRDX6

**Figure 3:**
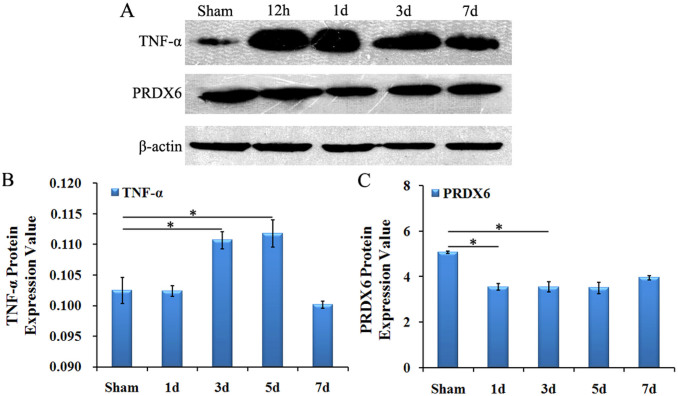
The expression of TNF-α and PRDX6 protein in rats with cSCI. (A) Western blot showed the levels of TNF-α and PRDX6 in the spinal cord at 12 h, 1 d, 3 d, and 7 d after cSCI. β-actin is used as the loading control. (B) The density of TNF-α bands normalised to the β-actin control. The expression of TNF-α protein was significantly increased at 3 dpo and 5 dpo. (C) The density of PRDX6 bands normalised to the β-actin control. The level of PRDX6 protein was significantly decreased at 1 dpo and 3 dpo. Values are expressed in mean ± SED * p < 0.05 *vs.* sham group.

Using western blotting, we measured whether there was a significant change in protein expression of TNF-α and PRDX6 in spinal cord grey matter after cSCI. The results indicated that protein expression of TNF-α was significantly increased on 1 dpo and then decreased on 3 dpo, but the value was still higher than in the sham group (P < 0.05, Fig. 3A,B). However, the variation of PRDX6 was contrary to TNF-α, PRDX6 continuously declined after cSCI, particularly, dropping to the lowest level at 6 h and slightly increased 14 dpo (Fig. 3A,C).

### Lentivirus-mediated inhibition of TNF-α promotes motor function recovery by the up-regulation of PRDX6

**Figure 4:**
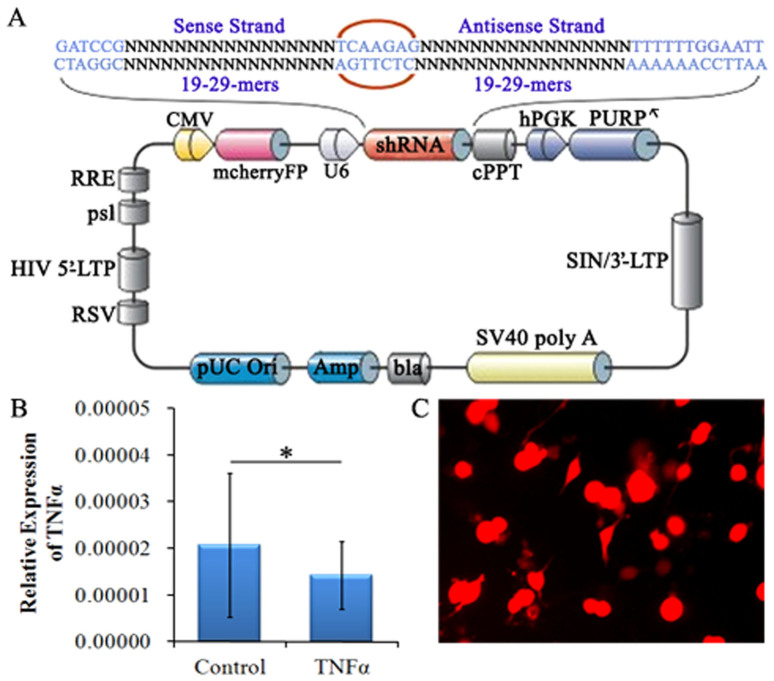
Preparation of TNF-α-RNAi-LV recombinants. (A) The schematic of TNF-α-RNAi-LV. Enhanced red fluorescent protein (mcherryFP) as a reporter gene was inserted in the plasmid. The framework also contains the antibiotic ampicillin and pUC Ori promoters for vector expression. (B) qRT-PCR shows effective interference of fragment for TNF-α inhibition, the expression of TNF-α were reduced. (C) Fluorescent image of TNF-α-RNAi-LV transfected into 293T cells.

Before the recombinant TNF-α interference lentivirus was transduced into the spinal cord, we examined the success of lentiviral targeting. The target of the TNF-α-RNAi-LV sequence was transfected into 293T cell line. qRT-PCR results showed that the TNF-α expression ratio decreased significantly (Fig. 4A,B).

After TNF-α-RNAi-LV was injected into the spinal cord, we confirmed its effect on the expression of TNF-α by immunofluorescent staining. Following lentivirus injection, horizontal sections of spinal cord tissue at the injection point were stained using immunofluorescence. The results showed expression of TNF-α in cells labelled by RFP (red), which suggested the TNF-α-RNAi-LV had successfully transfected host cells compared to those of the control group (Fig. 4C).

**Figure 5:**
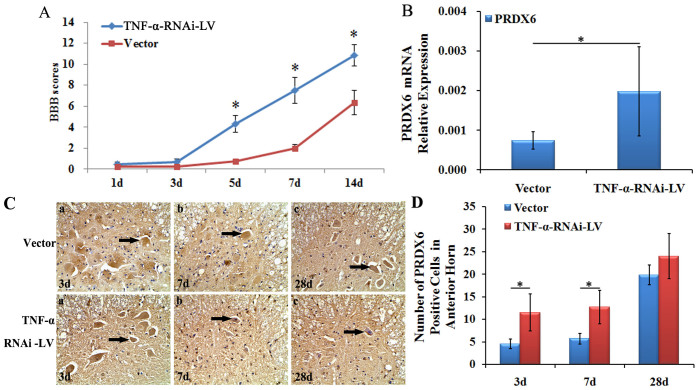
The change in behaviour and morphology in rats with cSCI after in vivo lentiviral TNF-α inference. (A) The BBB scores in the TNF-α-RNAi-LV group are significantly higher than vector group at 5 dpo, 7 dpo and 14 dpo. (B) The expression of PRDX6 mRNA is significantly up-regulated compared to vector group at 7 dpo. (C) Immunohistochemistry shows PRDX6-positive cells are located in the cytoplasm of neurons and glial cells. The PRDX6-positive cells are observed at 3 dpo, 7 dpo and 28 dpo. (D) The number of PRDX6-positive cells is significantly increased at 3 dpo and 7 dpo compared to vector group. Magnitude:40 × 10 μm. Values are expressed in mean ± SED. * p < 0.05 *vs.* vector group.

The BBB score after TNF-α-RNAi-LV administration was significantly higher than the vector group at 5 dpo, 7 dpo and 14 dpo (P < 0.05, Fig. 5A). The result revealed that inhibition of TNF-α could promote functional recovery in the hind limbs.

Moreover, after lentiviral-mediated TNF-α was transduced into the spinal cord, we confirmed that the suppression of TNF-α could up-regulate PRDX6 levels on Q-PCR. The results showed PRDX6 mRNA levels in the TNF-α-RNAi-LV group were significantly higher than that of the vector group after cSCI at 7 dpo (p < 0.05, Fig. 5B).

In addition, immunohistochemistry was used to visualise the expression of PRDX6 in the spinal cord. Compared to the TNF-α-RNAi-LV group, the number of PRDX6-positive cells in the vector group was significantly lower and was the lowest on 5 dpo (p < 0.05). The difference existed until 28 dpo (p < 0.05) after cSCI. However, when comparing the number of PRDX6-positive cells between the TNF-α-RNAi-LV group and the vector group, the TNF-α-RNAi-LV group had higher counts than the vector group at 3 dpo, 7 dpo and 28 dpo, and the number of PRDX6-positive cells were significantly different (p < 0.05, Fig. 5C, D).

## Discussion

In this study, we established a cSCI model in SD rats. The results of BBB scoring showed the rats had motor dysfunction after cSCI. We observed an up-regulation in the expression of TNF-α and a down-regulation in the expression of PRDX6 after cSCI as indicated via Q-PCR, WB and IHC. To explore the relationship between TNF-α and PRDX6, bioinformatics methods were used to predict the relationship between TNF-α and PRDX6. The bioinformatics analysis suggested that TNF-α and PRDX6 were both related to activating transcription factor 4 (ATF4). Therefore, it is assumed that TNF-α and PRDX6 were likely involved in certain cellular processes. To further verify this hypothesis, a series of experiments were conducted. The results of Q-PCR indicated that expression of TNF-α significantly increased and the expression of PRDX6 significantly decreased after the cSCI. However, the expression of PRDX6 was increased when lentiviral-mediated TNF-α RNAi vector was transduced into the spinal cord. These results suggested that inhibition of TNF-α can serve not only as a possible strategy for the treatment of SCI but also as an underlying mechanism related to the expression of both TNF-α and PRDX6.

### Lentivirus-mediated inhibition of TNF-α promotes motor function recovery in rat after SCI

In the present study, the effect of TNF-α on motor function recovery of SCI rats was observed. We evaluated the motor function of the rats via BBB scores and detected the expression of TNF-α using Q-PCR, IHC and WB. The results indicated an increase in TNF-α and a decrease of motor function after SCI, whereas lentivirus-mediated inhibition of TNF-α promoted motor function recovery in rats after SCI. This suggested that TNF-α is a predominant mediator in the spinal cord after injury. The results of TNF-α interference provided further indication of the role of TNF-α in the injured spinal cord, indicating its potential usage in future clinic trials.

TNF-α, as an anti-inflammatory factor, was associated with secondary injury after cSCI. TNF-α may aggravate the inflammatory response after cSCI and inhibit the recovery of spinal motor function as reported by numerous studies. TNF-α was increased rapidly after 1 hour in a model of traumatic spinal cord injury^24^. In addition, Hermann^25^ reported that TNF-α was released after SCI, which leads to central nervous system injury and secondary ischemia. After mice received intraspinal injection of quisqualic to create an excitotoxic injury, TNF-α mRNA in the spinal cord was increased at 3, 6, 12 and 24 h in the quisqualic-injected animals relative to the sham animals^26^. Additionally, inhibition of TNF-α can promote the recovery of limb motor function and may reduce the development of inflammation and tissue injury^27^. In addition, decreased TNF-α significantly alleviates neuropathic pain in the SCI model^28^. These reports support the view that TNF-α is related to the damage in motor function after SCI. The present study provided further evidence to support the role of TNF-α in cSCI by lentiviral-mediated TNF-α interference technology.

### TNF-α regulates the expression of PRDX6 to influence neuroplasticity

In the present work, we observed a decrease in PRDX6, which accompanied the increase of TNF-α after cSCI. As a result, it is assumed that a relationship between TNF-α and PRDX6 is probable. To illustrate the relationship between TNF-α and PRDX6, bioinformatics analysis was performed. The results of GeneMANIA analysis show that TNF-α and ATF4 are co-expressed, that PRDX6 and ATF4 are co-expressed, and that TNF-α and ATF3 are co-expressed. At the same time, ATF4 and ATF3 have physical interactions. Therefore, PRDX6 and TNF-α may have a direct relationship. M'Dahoma^29^ suggested that expression of TNF-α and ATF3 were increased to promote apoptosis in spinal cord injury. Another experiment reported that PRDX6 and ATF3 were co-expressed. Fatma^30^ found that the deficiency of PRDX6 led to increased levels of ROS and favours apoptosis and that ATF4 increased. To confirm the possible relationship between TNF-α and PRDX6 indicated by bioinformatics analysis, RNA interference (RNAi) experiments were performed. TNF-α-RNAi-LV resulted in the increase of PRDX6, which corresponded to the recovery of motor function in SCI rats. These results suggested that TNF-α influenced neuroplasticity, which may involve the regulation of PRDX6 expression.

Currently, there are a number of reports on the signalling pathway of TNF-α. Binding to the homotrimeric TNF-α receptor can activate three major signalling cascades, including caspase-8, JNK, and NF-κB^31^. It has been reported that the TNF-α trimer binds to the extracellular domain of TNFR1 to release the silencer of death domains (SODD) protein from the intracellular domain of TNFR1^32^. The intracellular domain of TNFR1 is bound by an adaptor protein, TNF receptor-associated death domain (TRADD)^33^, which recruits an additional adaptor protein, TNFR associated factor 2 (TRAF2)^34^. Consequently, the TNF-α-TRADD-TRAF2 complex can activate MEKKs, JNK and p38 MAPK^35^. Still, the cellular signalling of TNF-α was not well clarified. In this study, for the first time, the influence of TNF-α on neuroplasticity was shown to be related to PRDX6. As an antioxidant protein, PRDX6 can restore peroxidase catalysis and protect cells from oxidative injury^36^,^37^,^38^. Kümin^39^ reported that the lack of PRDX6 will aggravate inflammation and endothelial cell bleeding. The present findings indicated that TNF-α is indirectly connected with PRDX6. Moreover, we confirmed that TNF-α-RNAi-LV results in increased PRDX6 after cSCI, which suggested that TNF-α could regulate the expression of PRDX6. Therefore, PRDX6 may be a potential signalling molecule of TNF-α. In conclusion, TNF-α-RNAi-LV improved the motor function in the hind limbs of rats. The underlying mechanism may be related with the up-regulation of PRDX6. The present findings provide a new strategy for the clinical treatment of SCI in the future.

## Author Contributions

T.W. and X.Z. designed the experiments. X.Z. and L.S. wrote the main manuscript text. X.G. and D.J. prepared all figures. Z.Z. and T.L. conducted the western blot experiments. X.Z. and Y.R. worked the animal model. X.L., L.L. and X.H. conducted the behaviour tests. Q.X. and Z.Z. conducted the Q-PCR. X.H. and J.C. conducted the immunohistochemistry and immunofluorescence. All authors reviewed the manuscript.

## Figures and Tables

**Table 1:** Detail information on the selection of primers for real-time RT-PCR experiments

Name	Primers sequence(sense)	Primers sequence (antisense)
β-actin	GAAGATCAAGATCATTGCTCCT	TACTCCTGCTTGCTGATCCA
TNF-α	CAGAAATCAAGGAGCATTTG	CTGCTCCACTGCCTTGCTTT
PRDX6	GTGCGACAGGAACCAGAAGC	CTCGGGCTTCTGGTTCCTGT

## References

[b1] Garraway S. M. *et al.* Intermittent noxious stimulation following spinal cord contusion injury impairs locomotor recovery and reduces spinal brain derived neurotrophic factor tropomyosin receptor kinase signaling in adult rats. Neuroscience 199, 86−102 (2011). 22027236 10.1016/j.neuroscience.2011.10.007 PMC3237800

[b2] Wu J. *et al.* TrkB. T1 contributes to neuropathic pain after spinal cord injury through regulation of cell cycle pathways. J Neurosci 33, 12447−12463 (2013). 23884949 10.1523/JNEUROSCI.0846-13.2013 PMC3721848

[b3] Saville L. R. *et al.* A monoclonal antibody to CD11d reduces the inflammatory infiltrate into the injured spinal cord: a potential neuroprotective treatment. J Neuroimmunol 156, 42−57 (2004). 15465595 10.1016/j.jneuroim.2004.07.002

[b4] Penas C. *et al.* Spinal cord injury induces endpodpolasmic reticulum stress with different cell type dependent response. J Neurochem 102, 1242−1255 (2007). 17578450 10.1111/j.1471-4159.2007.04671.x

[b5] Chi L. Y. *et al.* The dual role of tumor necrosis factor-alpha in the pathophysiology of spinal cord injury. NeurosciLett 438, 174−179 (2008). 10.1016/j.neulet.2008.04.043 18468795

[b6] Willemen H. L. *et al.* Microglial/macrophage GRK2 determines duration of peripheral IL-1beta induced hyperalgesia: contribution of spinal cord CX3CR1, p38 and IL-1 signaling. Pain 150, 550−560 (2010). 20609517 10.1016/j.pain.2010.06.015 PMC3099597

[b7] Boato F. *et al.* Absence of IL-1beta positively affects neurological outcome, lesion development and axonal plasticity after spinal cord injury**. J Neuroinflammation 10, 6 (2013). 23317037 10.1186/1742-2094-10-6 PMC3585738

[b8] Guptarak J. *et al.* Inhibition of IL-6 signaling: A novel therapeutic approach to treating spinal cord injury pain. Pain 154, 1115−1128 (2013). 23639820 10.1016/j.pain.2013.03.026

[b9] Tzeng S. F. *et al.* Tumor necrosis factor-alpha regulation of the Id gene family in astrocytes and microglia during CNS inflammatory injury. Glia 26, 139−152 (1999). 10384879 10.1002/(sici)1098-1136(199904)26:2<139::aid-glia5>3.0.co;2-1

[b10] Ohtori S. *et al.* TNF-α and TNF-α receptor type 1 upregulation in glia and neurons after peripheral nerve injury: studies in murine DRG and spinal cord. Spine 29, 1082−1088 (2004). 15131433 10.1097/00007632-200405150-00006

[b11] Kawasaki Y. *et al.* Cytokine mechanisms of central sensitization: distinct and overlapping role of interleukin-1 beta, interleukin-6, and tumor necrosis factor-alpha in regulating synaptic and neuronal activity in the superficial spinal cord**. J Neurosci 28, 5189−5194 (2008). 18480275 10.1523/JNEUROSCI.3338-07.2008 PMC2408767

[b12] Guadagno J. *et al.* Microglia-derived TNF alpha induces apoptosis in neural precursor cells via transcriptional activation of the Bcl-2 family member Puma. Cell Death Dis 4, 538 (2013). 10.1038/cddis.2013.59 PMC3613837 23492769

[b13] Xia M. & Zhu Y. Zhu FOXO3a involvement in the release of TNF-α stimulated by ATP in spinal cord astrocytes. J MolNeurosci 51, 792−804 (2013). 10.1007/s12031-013-0067-8 23860688

[b14] Cortez M. *et al.* A high-fat diet increases IL-1, IL-6, and TNF-α production by increasing NF-kappa B and attenuating PPAR-gamma expression in bone marrow mesenchymal stem cells. Inflammation 36, 379−386 (2013). 23079940 10.1007/s10753-012-9557-z

[b15] Walsh B. *et al.* Over expression of Prdx6 and resistance to peroxide induced death in Hepa1-6 cells: Prdx suppression increases apoptosis. Redox Rep 14, 275−284 (2009). 20003713 10.1179/135100009X12525712409652

[b16] Zhou S. *et al.* Functional interaction of glutathione S-transferase pi and peroxiredoxin 6 in intact cells. Int J Biochem Cell Biol 45, 401−407 (2013). 23164639 10.1016/j.biocel.2012.11.005 PMC3555408

[b17] Brown S. D. & Brown L. A. Ethanol (EtOH) induced TGF-beta1 and reactive oxygen species production are necessary for EtOH induced alveolar macrophage dysfunction and induction of alternative activation. Alcohol ClinExp Res 36, 1952−1962 (2012). 10.1111/j.1530-0277.2012.01825.x PMC3414680 22551312

[b18] Manevich Y. *et al.* Structure and phospholipase function of peroxiredoxin 6: identification of the catalytic triad and its role in phospholipid substrate binding. J Lipid Res 48, 2306−2318 (2007). 17652308 10.1194/jlr.M700299-JLR200

[b19] Kim S. Y. *et al.* H2O2-dependent hyperoxidation of peroxiredoxin 6 (Prdx6) plays a role in cellular toxicity via up-regulation of iPLA2 activity. J BiolChem 283, 33563−33568 (2008). 10.1074/jbc.M806578200 PMC2662274 18826942

[b20] Sorokina E. M. *et al.* Intracellular targeting of peroxiredoxin 6 to lysosomal organelles requires MAPK activity and binding to 14-3-3epsilon. Am J Physiol Cell Physiol 300, C1430−1441 (2011). 21346153 10.1152/ajpcell.00285.2010 PMC3118628

[b21] Fatma N. *et al.* Peroxiredoxin 6 delivery attenuates TNF-α and glutamate induced retinal ganglion cell death by limiting ROS levels and maintaining Ca2+ homeostasis. Brain Res 1233, 63−78 (2008). 18694738 10.1016/j.brainres.2008.07.076 PMC3488878

[b22] Gallagher B. M. & Phelan S. A. Investigating transcriptional regulation of Prdx6 in mouse liver cells. Free RadicBiol Med 42, 1270−1277 (2007). 10.1016/j.freeradbiomed.2007.01.023 17382207

[b23] Basso D. M. *et al.* A sensitive and reliable locomotor rating scale for open field testing in rats. J Neurotrauma 12, 1−21 (1995). 7783230 10.1089/neu.1995.12.1

[b24] Wang C. X. *et al.* Expression of tumor necrosis factor and its mRNA in the spinal cord following a weight-drop injury. Neuroreport 13, 1391−1393 (2002). 12167759 10.1097/00001756-200208070-00008

[b25] Hermann G. E. *et al.* Tumor necrosis factor-a induces FOS and strongly potentiates glutamate-mediated cell death in the rat spinal cord. Neurobiol Dis 8, 590−599 (2001). 11493024 10.1006/nbdi.2001.0414

[b26] Brewer K. L. & Nolan T. A. Spinal and supraspinal changes in tumor necrosis factor a expression following excitotoxic spinal cord injury. J MolNeurosci 31, 13−21 (2007). 10.1007/BF02686114 17416966

[b27] Genovese T. *et al.* TNF-α blockage in a mouse model of SCI: evidence for improved outcome. Shock 29, 32−41 (2008). 17621255 10.1097/shk.0b013e318059053a

[b28] Peng X. M. *et al.* Tumor necrosis factor a contributes to below level neuropathic pain after spinal cord injury. Ann Neurol 59, 843−851 (2006). 16634039 10.1002/ana.20855

[b29] M'Dahoma S. *et al.* Spinal cord transection-induced allodynia in rats--behavioral, physiopathological and pharmacological characterization. PloS One 14, e102027 (2014). 25019623 10.1371/journal.pone.0102027 PMC4096923

[b30] Fatma N. *et al.* Deficiency of Prdx6 in lens epithelial cells induces ER stress response-mediated impaired homeostasis and apoptosis. Am J Physiol Cell Physiol 301, C954−967(2011). 21677259 10.1152/ajpcell.00061.2011 PMC3191562

[b31] Park K. M. & Bowers W. J. Tumor necrosis factor a mediated signaling in neuronal homeostasis and dysfunction. Cell Signal 22, 977−983 (2010). 20096353 10.1016/j.cellsig.2010.01.010 PMC2860549

[b32] Parameswaran N. & Patia S. Tumor necrosis factor a signaling in macrophages. Crit Rev Eukaryot Gene Expr 20, 87−103l (2010). 21133840 10.1615/critreveukargeneexpr.v20.i2.10 PMC3066460

[b33] Hsu H. *et al.* The TNF receptor 1-associated protein TRADD signals cell death and NF-kappa B activation. Cell 81, 495−504 (1995). 7758105 10.1016/0092-8674(95)90070-5

[b34] Takeuchi M. *et al.* Anatomy of TRAF2. Distinct domains for nuclear factor-kappaB activation and association with tumor necrosis factor signaling proteins. J BiolChem 271, 19935−19942 (1996). 10.1074/jbc.271.33.19935 8702708

[b35] Ichijo H. *et al.* Induction of apoptosis by ASK1, a mammalian MAPKKK that activates SAPK/JNK and p38 signaling pathways. Science 275, 90−94 (1997). 8974401 10.1126/science.275.5296.90

[b36] Kubo E. *et al.* Protein expression profiling of lens epithelial cells from Prdx6 depleted mice and their vulnerability to UV radiation exposure. Am J Physiol Cell Physiol 298, C342−354 (2010). 19889963 10.1152/ajpcell.00336.2009 PMC2822493

[b37] Fatma N. *et al.* Impaired homeostasis and phenotypic abnormalities in Prdx6-/-mice lens epithelial cells by reactive oxygen species: increased expression and activation of TGF beta. Cell Death Differ 12, 734−750 (2005). 15818411 10.1038/sj.cdd.4401597

[b38] Chhunchha B. *et al.* Specificity protein, Sp1-mediated increased expression of Prdx6 as a curcumin induced antioxidant defense in lens epithelial cells against oxidative stress. Cell Death Dis 2, 234 (2011). 10.1038/cddis.2011.121 PMC3223701 22113199

[b39] Kumin A. *et al.* Peroxiredoxin 6 is required for blood vessel integrity in wounded skin. J Cell Biol 179, 747−760 (2007). 18025307 10.1083/jcb.200706090 PMC2080929

